# Trips-Viz: an environment for the analysis of public and user-generated ribosome profiling data

**DOI:** 10.1093/nar/gkab323

**Published:** 2021-05-05

**Authors:** Stephen J Kiniry, Ciara E Judge, Audrey M Michel, Pavel V Baranov

**Affiliations:** School of Biochemistry and Cell Biology, University College Cork, Cork, Ireland; School of Biochemistry and Cell Biology, University College Cork, Cork, Ireland; School of Biochemistry and Cell Biology, University College Cork, Cork, Ireland; Ribomaps Ltd, Western Gateway Bld, Western Rd, Cork, Ireland; School of Biochemistry and Cell Biology, University College Cork, Cork, Ireland; Shemyakin-Ovchinnikov Institute of Bioorganic Chemistry, RAS, Moscow, Russia

## Abstract

Trips-Viz (https://trips.ucc.ie/) is an interactive platform for the analysis and visualization of ribosome profiling (Ribo-Seq) and shotgun RNA sequencing (RNA-seq) data. This includes publicly available and user generated data, hence Trips-Viz can be classified as a database and as a server. As a database it provides access to many processed Ribo-Seq and RNA-seq data aligned to reference transcriptomes which has been expanded considerably since its inception. Here, we focus on the server functionality of Trips-viz which also has been greatly improved. Trips-viz now enables visualisation of proteomics data from a large number of processed mass spectrometry datasets. It can be used to support translation inferred from Ribo-Seq data. Users are now able to upload a custom reference transcriptome as well as data types other than Ribo-Seq/RNA-Seq. Incorporating custom data has been streamlined with RiboGalaxy (https://ribogalaxy.ucc.ie/) integration. The other new functionality is the rapid detection of translated open reading frames (ORFs) through a simple easy to use interface. The analysis of differential expression has been also improved via integration of DESeq2 and Anota2seq in addition to a number of other improvements of existing Trips-viz features.

## Graphical Abstract

**Graphical Abstract:**
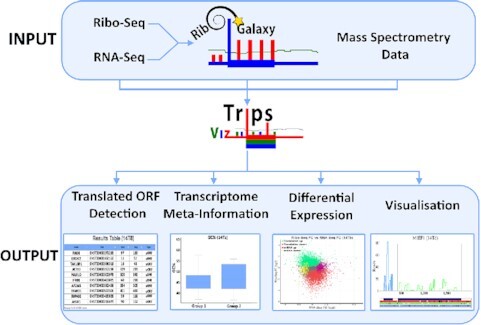
Trips-Viz enables numerous analyses of public and user-generated ribosome profiling and proteomics data.

## INTRODUCTION

Ribosome profiling (Ribo-Seq) is a technique that allows for large scale isolation of mRNA fragments which are being protected by actively translating ribosomes (1). These fragments can then be mapped to a genome or transcriptome and utilized in a number of different ways. This includes detection of novel translated open reading frames and pause sites, as well as identification of differentially translated genes, for reviews see (2–4). To date there has been a number of different software packages created to explore each of these aspects of ribosome profiling (5). Many of these require some computational expertise and familiarity with command line usage. In addition, specific expertise and time are required to process and map the raw ribosome profiling reads. This too has been addressed by many packages (6–10), which aim to simplify the task of processing ribosome profiling data. Furthermore, many databases now exist which provide pre-processed publicly available ribosome profiling data (11–15), allowing users to carry out analysis either explicitly or implicitly through visualization of the data.

Among these is Trips-Viz, a transcriptome analysis platform with a focus on visualization and analysis of processed Ribo-Seq and RNA-Seq data. The triplet periodicity of ribosome profiling data allows for the detection of the translated reading frame (16). Trips-Viz takes advantage of this by colour coding Ribo-Seq reads according to the supported reading frame. This facilitates users to rapidly view Ribo-Seq profiles aggregated from numerous studies, providing the functionality to visually decipher not just the location but also the reading frame where translation is most likely occurring for a given mRNA transcript. Other capabilities include the option to directly compare multiple datasets on a single mRNA transcript, the functionality to carry out differential expression/translation analysis and calculate and visualize simple meta data statistics for individual datasets such as the distribution of read lengths, strength of triplet periodicity and metagene profiles. These statistics are useful for assessing data quality. Thus, Trips-Viz provides users with a large amount of relevant information which they can obtain very quickly and without the need for computational expertise and resources. Here we will discuss the major updates to Trips-Viz since its original publication (17) focusing on its server functionality. For a full list of updates see https://trips.ucc.ie/stats/.

## NEW AND ENHANCED FEATURES

### Improved ease of use

Since the launch of Trips-Viz users were able to process and upload their own files to be viewed privately and shared with collaborators. However, this required some familiarity with the command line and was a complex process. As one of the goals of Trips-Viz is to reduce users’ computational workload, this was not an ideal solution. To address this, the relevant scripts were streamlined and incorporated into RiboGalaxy (6). RiboGalaxy is a GUI based platform made for processing Ribo-Seq and RNA-Seq data, based on the Galaxy platform (18), designed to streamline and standardize analysis of biological data while making the process transparent and reproducible. Users can now easily carry out all the steps necessary to process a raw fastq file for uploading to Trips-Viz, all within RiboGalaxy. This includes upload of custom transcriptomes to Trips-Viz, a feature that was absent at the time of the Trips-Viz launch. Thus, Trips-Viz can now be used for data obtained from any species irrespective of whether a corresponding transcriptome is already available. The custom transcriptomes can also be processed using RiboGalaxy if users upload the relevant transcriptome fasta and GTF files.

While comprehensive help pages have been available from the beginning (https://trips.ucc.ie/help/), Trips-Viz is a GUI based tool which makes it difficult to easily explain the steps needed to carry out certain analyses when compared to a command line tool. This, coupled with the growing functionality and diversity of Trips-Viz visualizations makes using it more daunting for new users. To address this, videos have been embedded in the help pages, one for each plot type. Videos walk new users through the use of each plot, explaining the meanings of various settings and parameters and effects that they make on a specific visualization. This makes it easier for new users to quickly become familiar with the Trips-Viz interface and use it to its full potential. Users can now also download most plots on Trips-Viz in high resolution in .png format in addition to having more control over the size and colour of different plot elements on the settings page.

### Mass spectrometry data

Trips-viz was originally designed solely for the analysis and visualization of Ribo-Seq and RNA-Seq data. Since then, we have expanded it to incorporate other data types, primarily mass spectrometry data. A popular application of Ribo-Seq data is to look for evidence of translation outside of regions annotated as protein-coding (19,20). As mass spectrometry data also provide information on translation, it is reasonable to conclude that interrogating both types of data simultaneously can be greatly beneficial (13,21–23). While there are numerous useful resources to explore publicly available mass spectrometry data (24,25), many look only for support from existing annotated CDS’s, diminishing their usefulness in terms of providing supporting information to Ribo-Seq findings. In Trips-Viz we do not limit the peptide search to CDS ORFs, opting instead to search all 3 reading frames across the entire transcript. This is done for all principal (26) transcript isoforms in the transcriptome. This enables us to find proteomics support for translated regions regardless of location within the transcript and leverage the same graphs and colour scheme used to display Ribo-Seq data allowing users to easily see the frame and location of detected peptides.

**Figure 1.**
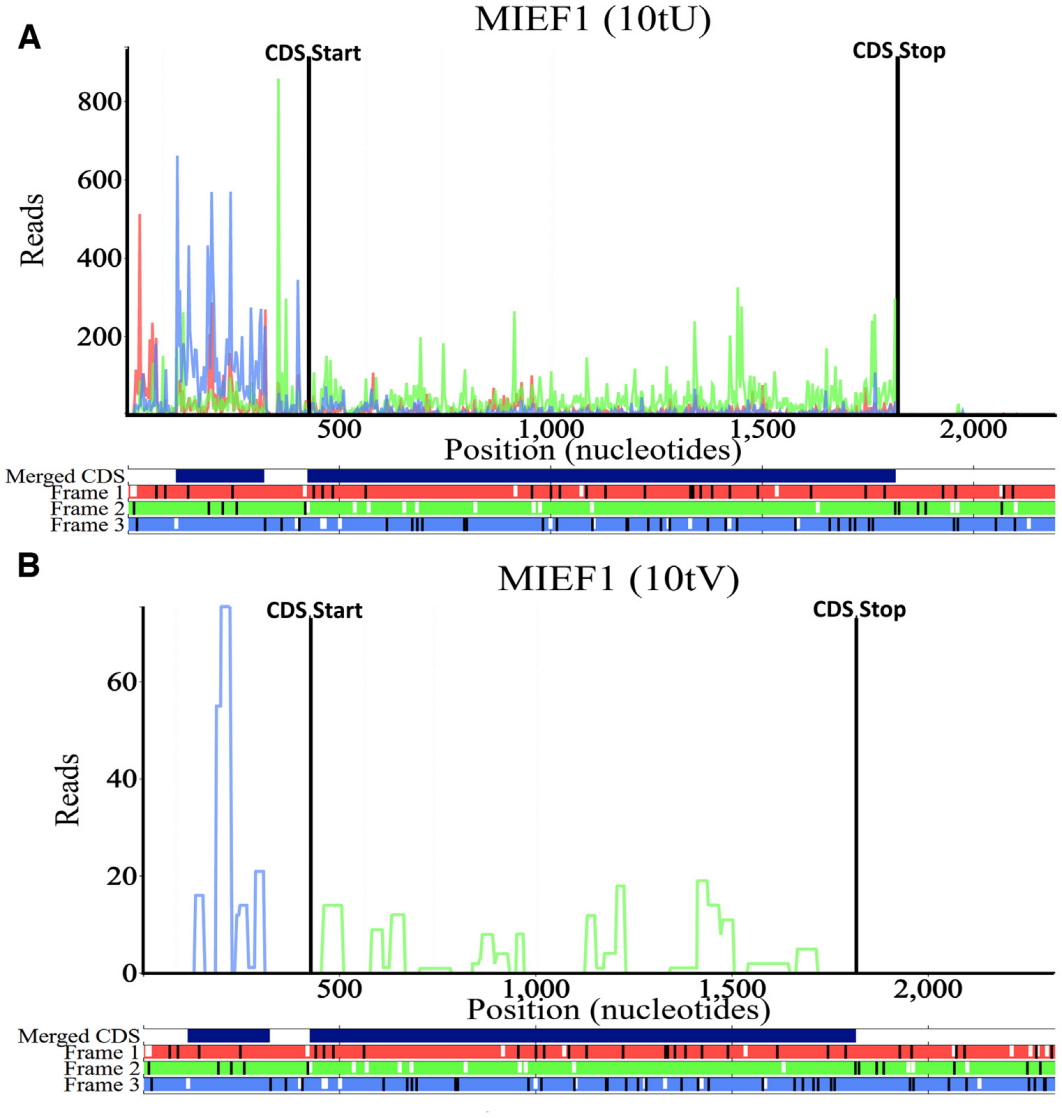
The Trips-Viz single transcript plot for the human gene *MIEF1* principal isoform (Transcript ENST00000325301) (zoomed in to show the 5′ Leader/CDS). (**A**) An aggregate of Ribo-Seq reads from multiple studies. The triplet periodicity of the Ribo-Seq reads clearly shows a bias towards the second reading frame (green) which matches the reading frame of the annotated CDS. Ribo-Seq reads are mostly but not wholly confined to the CDS. Atypically there are many reads present in the 5′ leader which are biased towards the third reading frame (blue) matching the location of an ORF in the third reading frame. This is corroborated by the proteomics data in panel (**B**). Locations encoding peptides from an aggregate of mass spectrometry datasets are displayed. All peptides bar one in the 3′ trailer (not shown) are found either within the CDS (frame 2) or in the third reading frame matching the position of the uORF in the 5′ leader. The code in brackets in the title of the plot can be used to generate the profile in a browser, e.g. following this link https://trips.ucc.ie/short/10tU will load the plot shown in panel A, for more information on short codes see the Trips-Viz help pages.

To date there are 3152 processed mass spectrometry datasets available on Trips-Viz. The pipeline for Trips-Viz proteomics data integration involves searching for peptides in all three reading frames using MSFragger (27), then removing peptides with an FDR >1% using Philosopher (28). The output is then parsed and results are uploaded to Trips-Viz, where they are coloured according to the matching reading frame, in a similar manner to Ribo-Seq data. Their visualization can then be used to find novel translated ORFs, or to corroborate results observed from Ribo-seq data. See an example in Figure 1 where peptides from a uORF can be seen for the human gene *MIEF1*, which has previously been shown to be translated and was predicted to code for a functional protein (29). Subsequently, its product was identified as a part of protein complex involved in assembly of mitochondrial ribosome (30) and further evidence supported its function in mitochondrial translation (31). The proteomics data also suggest that the product encoded by the *MIEF1* uORF is the main product of its mRNA (29,32), while the synthesis of the MIEF1 protein is activated by stress conditions. Additionally, users can now also upload custom mappings of mass spectrometry data to Trips-Viz.

### Detecting non-canonical Ribo-Seq signals

Detecting translated open reading frames (ORFs) using ribosome profiling data has been a subject of much interest in recent years. While many different programs now exist that can detect translated ORFs (20,33–40), the majority of them require bioinformatic expertise as well as processed Ribo-Seq data, both of which may be expensive to acquire in terms of time and computational power. Trips-viz is now capable of automatically detecting Ribo-Seq signals outside annotated CDS regions in a simple but effective manner using previously processed Ribo-Seq data. This allows users to quickly and easily use an aggregate of data from multiple studies with good periodicity which can dramatically improve detection.

Trips-viz differs somewhat in its approach from most existing translated ORF detection approaches. It does not use machine learning methods as these rely on the availability of a ‘gold-standard’ set of translated ORFs, which can be difficult to achieve even in well annotated organisms. Instead, at present, Trips-Viz first discards all read-lengths with weak triplet periodicity and then extracts three to four Ribo-Seq features (depending on the region of interest) from ORFs and ranks these features individually from strongest translational signal to weakest. These features include the increase of Ribo-Seq density at the start codon, the drop in Ribo-Seq density at the stop codon, the difference in in-frame and out-of-frame Ribo-Seq reads and the number of codons in the region of interest where the in-frame reads are higher than the out-of-frame reads. These individual ranks are then aggregated to determine a global rank for every ORF. It will then display a list of ORFs from strongest to weakest Ribo-Seq signal. This simplistic method does not allow for binary classification of ORFs as translated/untranslated as many other programs do. However, the goal of Trips-Viz differs in that it aims to allow users to rapidly find *individual* examples of high confidence non-canonical translation via manual inspection that warrant deeper investigation.

**Figure 2.**
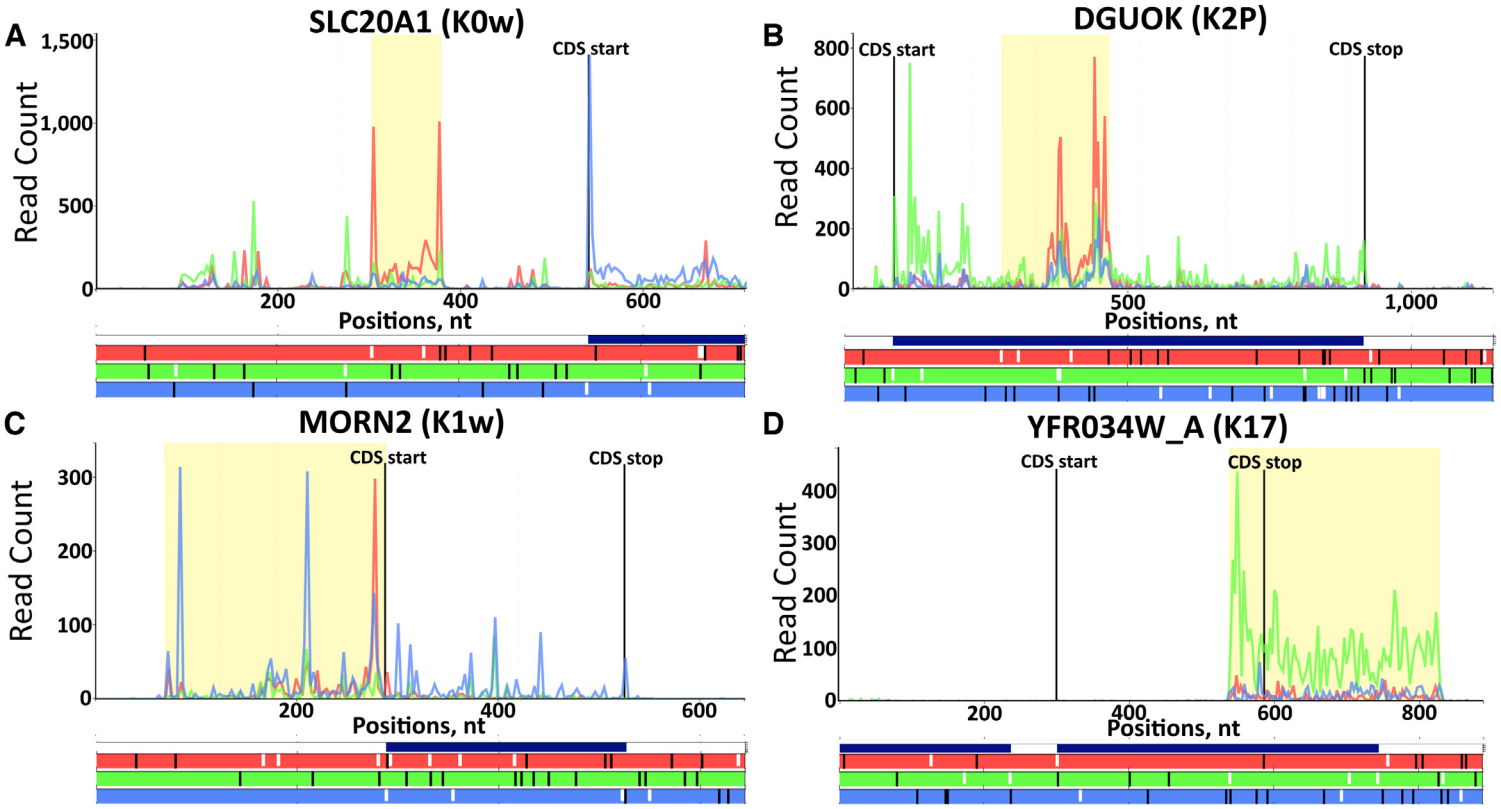
Examples of highly ranked ORFs detected by Trips-Viz. At the bottom of each plot the open reading frame architecture is represented with three horizontal bars coloured red, green and blue to display each of the three reading frames. AUG codons are denoted by short white lines while stop codons are denoted by longer black lines. The CDS start and CDS stop positions are shown with the vertical black lines on the main plot along with counts of Ribo-Seq reads displayed in red, green and blue matching each of the three reading frames beneath. The ‘merged CDS’ bar above the reading frame bars displays a union of all annotated CDS regions in the corresponding locus. (**A**) An AUG-initiated uORF of the human gene *SLC20A1* (Transcript ENST00000272542) in frame 1 (the annotated CDS is in frame 3). (**B**) A nested ORF in frame 1 of the human gene *DGUOK* (Transcript ENST00000264093) where the annotated CDS is in frame 2. (**C**) An N-terminal extension of the mouse gene Morn2 (Transcript ENSMUST00000061703). (**D**) An overlapping downstream ORF of the yeast gene *YFR034W_A*. Here the merged CDS bar extends into the 3′ trailer of *YFR034W_A* indicating the presence of another gene at the same locus. In this case the gene in question is *YFR035C* which is transcribed from the opposite strand, so it cannot explain the Ribo-Seq reads in the 3′ trailer of *YFR034W_A*. In addition, the reads extend beyond the merged CDS bar.

To aid in this manual inspection, results are displayed in the form of a table showing the top 1000 ranked ORFs, with the option of downloading the entire table. Each ORF will have a link allowing the user to view the ORF in question in the corresponding transcript with the selected data, allowing users to rapidly visualize each ORF using only the datasets they selected, see Figure 2. Translation of ORFs that belong to noncoding RNAs can be detected in addition to ORFs from annotated coding transcripts which are broken down into the following categories depending on their location relative to the CDS: upstream ORFs, overlapping upstream ORFs, nested ORFs, downstream ORFs and n-terminal extensions.

### Differential expression/translation

**Figure 3.**
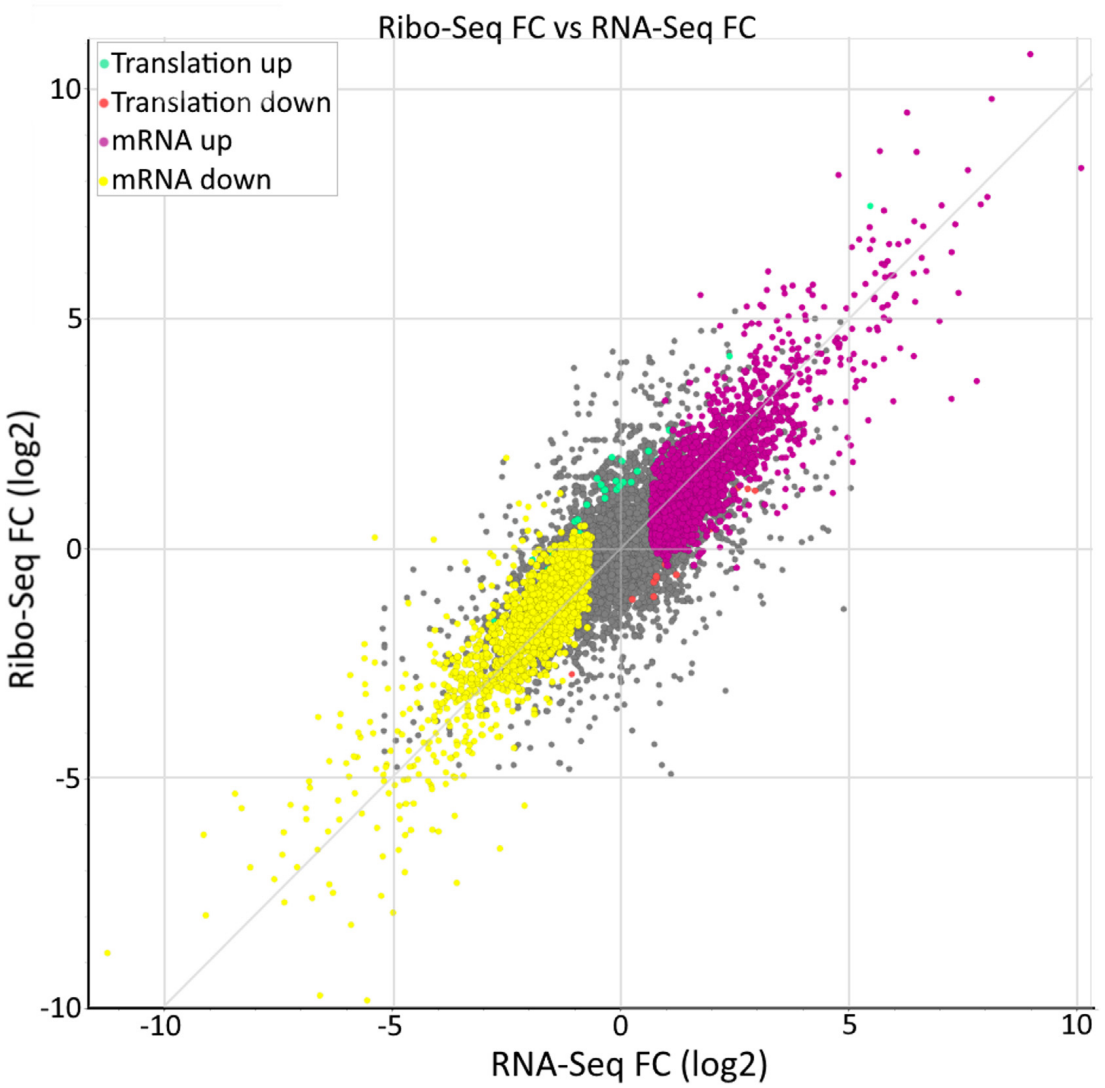
An example of the differential gene expression analysis using DESeq2, using data from Iwasaki *et al.* (52). Genes whose expression did not change significantly are coloured grey, translationally upregulated/downregulated genes are in green/red, while changes in mRNA levels are in purple/yellow. Hovering over any of the points on the plot triggers a pop-up window with information specific to the corresponding gene/transcript and fold changes. Clicking on any of the points invokes a separate tab showing the comparison plot for ribosome footprints mapped to the corresponding gene.

Since its launch, Trips-Viz provided a single option for carrying out differential expression analysis on principal transcript isoforms using the z-score transformation (29). While this performs adequately, there are more accurate and powerful approaches for this purpose (41). To this end two new options were incorporated into Trips-Viz, DESeq2 (42) and anota2seq (43), which will allow users to quickly compare the results across the three methods. An example plot can be seen in Figure 3, showing the Ribo-Seq fold change versus the RNA-Seq fold change. It allows users to quickly see expression of which genes are affected at the RNA and/or translation levels. Similarly to the *z*-score plot, users can click on any point in the plot to invoke a comparison plot where footprint densities are compared for two conditions for the corresponding transcript. Users can also download the inputs and outputs of DESeq2 and anota2seq for further exploration. It is recommended that DESeq2 and anota2seq be used over the *z*-score method, however these require a minimum of two and three replicates respectively, thus the z-score transformation approach remains the only option for exploring datasets lacking replicates which could be useful during preliminary data generation and pilot experiments.

### Transcriptome metainformation

**Figure 4.**
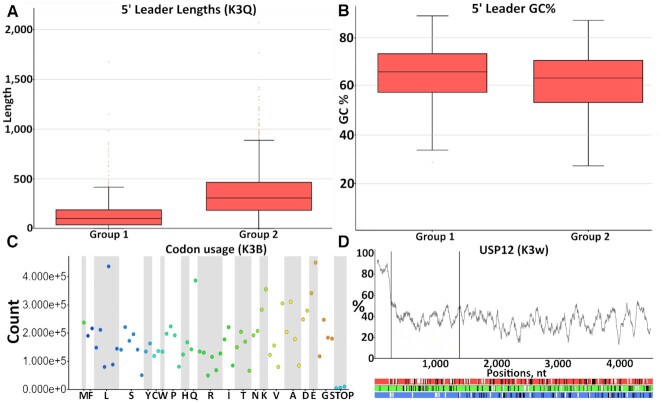
Examples of the types of plots generated from the transcriptome info page. (**A**) A comparison of 5′ leader lengths and (**B**) 5′ leader GC% between upregulated/downregulated genes in response to RocA treatment (52). (**C**) The codon usage occurrence within the CDS of all principal transcript isoforms in the human transcriptome using Gencode v25 (53). (**D**) The GC content of the human gene *USP12*. The same visualization can be used for individual nucleotide frequencies within sliding windows or for minimum free energy of potential RNA secondary structures as calculated by ViennaRNA (54).

A new section has been added to Trips-Viz to address all queries not directly related to Ribo-seq/RNA-Seq or other data types. This can be used to address simple questions about a transcriptome such as how many genes/transcripts are annotated and how many are coding/non-coding, what is the codon usage in CDS regions or what is the difference in GC content in 5′ leaders (commonly known as 5′ UTR’s) versus 3′ trailers (commonly known as 3′ UTR’s). It can also be used to retrieve nucleotide sequences of some or all transcripts, either in their entirety or for specific subsections (5′ leader, CDS, 3′ trailer). However, most plots on this page can be generated using subsets of transcripts. This can be used to gain a deeper understanding of differential expression/translation results, for example, by comparing these features between groups of upregulated and downregulated genes. An example is presented in Figure 4.

### Comparison with other tools

It has now been over a decade since the introduction of the ribosome profiling technique and in that time a plethora of different tools have been developed that cover almost every aspect of ribosome profiling data analysis (5). Carrying out a detailed analysis against all available tools would be difficult due to the sheer number of them. Instead, these have been broadly split into two categories. There now exists many offline tools such as Plastid (8), RiboProfiling (44), riboflow (9) and ribotaper (20) to name just a few, which are designed to be downloaded and installed locally for users to process and analyze their own data. These tools have considerable overlap with Trips-Viz in terms of the type of analysis that they provide but as these tools typically require some computational expertise the target audience differs from Trips-Viz which aims to provide a solution to those without such expertise. Instead, a more detailed comparison was made to other online databases which either provide pre-processed data or provide an easy way to process Ribo-Seq data which does not require computational expertise. These tools include RiboToolKit (7), SmProt (45), HRPDViewer (46), TranslatomeDB (15), RiboViz (47), RPFdb (12), OpenProt (13), GWIPS-Viz (48), RiboGalaxy (6) and RiboStreamR (49). The features of these tools are listed in Table 1.

**Table 1.** Comparison table showing the presence (tick) or absence (x) of various features (rows) available in Trips-Viz and similar tools (columns)

	*Trips-Viz*	*RiboToolKit*	*SmProt*	*HRPDViewer*	*TranslatomeDB*	*RiboViz*	*RPFdb*	*OpenProt*	*GWIPS-VIZ*	*RiboGalaxy*	*RibostreamR*
Web-Upload	✓	✓	✗	✗	✓	✗	✗	✓	✓	✓	✓
Batch-Uploading	✓	✓	✗	✗	✗	✗	✗	✓	✗	✗	✓
Local Install	✓	✓	✗	✗	✗	✓	✗	✗	✗	✓	✗
Pre-processed data	✓	✗	✓	✓	✓	✓	✓	✓	✓	✗	✗
Contamination checking	✓	✓	✗	✗	✗	✗	✗	✗	✗	✓	✓
Quality checking	✓	✓	✗	✗	✗	✓	✓	✗	✓	✓	✓
RPF visualisation	✓	✓	✗	✓	✓	✓	✓	✗	✓	✓	✓
RNA-Seq visualisation	✓	✗	✗	✗	✓	✓	✗	✗	✓	✓	✓
Mismatch detection	✓	✗	✗	✗	✗	✗	✗	✗	✗	✗	✗
Visualization of subcodon profiles	✓	✗	✗	✗	✗	✗	✗	✗	✗	✓	✓
Nucleotide sequence retrieval	✓	✗	✓	✗	✗	✗	✗	✗	✓	✓	✗
Codon Occupancy	✗	✓	✗	✗	✗	✗	✗	✗	✓	✓	✗
Pause detection	✗	✓	✗	✗	✗	✗	✗	✗	✗	✗	✗
Codon Frequency	✓	✓	✗	✗	✗	✗	✗	✗	✗	✗	✗
Meta-Codon plots	✗	✓	✗	✗	✗	✗	✗	✗	✗	✗	✗
mRNA expression	✓	✓	✗	✗	✓	✓	✗	✗	✗	✓	✓
RPF expression	✓	✓	✗	✓	✓	✓	✓	✗	✗	✓	✓
Translation efficiency analysis	✓	✓	✗	✗	✓	✓	✗	✗	✗	✓	✓
Differential translation analysis	✓	✓	✗	✗	✓	✗	✗	✗	✗	✓	✓
Translated ORF detection	✓	✓	✓	✗	✗	✗	✓	✓	✗	✗	✗
Proteomics Analysis	✓	✗	✓	✗	✗	✗	✗	✓	✗	✗	✗
GO/Pathway analysis	✗	✓	✗	✗	✓	✗	✗	✗	✗	✗	✗
MetaGene Plots	✓	✓	✗	✗	✗	✓	✓	✗	✗	✓	✓
Reproducibility between replicates	✓	✗	✗	✗	✓	✗	✗	✗	✗	✗	✗

While this table attempts to capture the main differences between Trips-Viz and similar tools it is difficult to simplify all differences into simple binary categories. To that end we discuss a specific example which shows various features of Trips-Viz which can be used in concert to investigate the translation of specific RNA transcripts and quickly make interesting biological observations.

**Figure 5.**
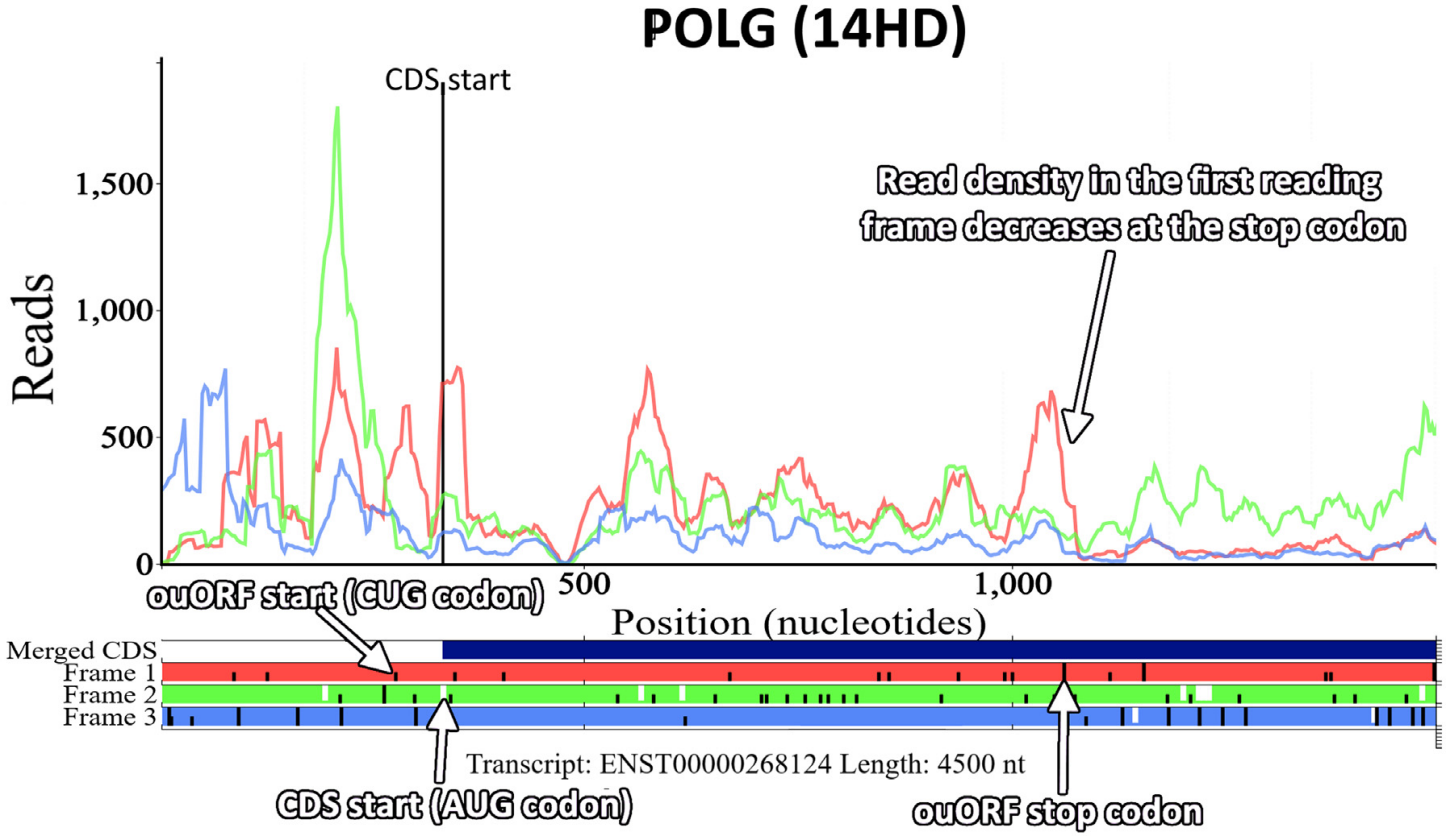
The Trips-Viz single transcript plot for the human gene *POLG* (transcript ENST00000268124) using an aggregate of Ribo-Seq reads from multiple studies. The view has been zoomed in around the annotated CDS start. There is a Ribo-seq bias within the first reading frame (red) across the entirety of the ouORF supporting the translation of the correct frame. The ORF architecture beneath the plot (horizontal red, green and blue bars), shows the positions of AUG codons (short white lines), stop codons (longer black lines) and CUG codons (short black lines), making it easier to see the start and stop positions of the ouORF. The third CUG codon in the first reading frame is the translation initiation site of the ouORF (50,51), which has been highlighted in the figure.

The human gene *POLG* has been recently shown to encode an additional protein in an overlapping upstream open reading frame (ouORF) (50,51). Visualizing the translation of the *POLG* mRNA using currently available public Ribo-Seq data in Trips-Viz makes the translation of the ouORF clear due to a number of features (Figure 5). Most important is the ability to visualize subcodon profiles by colouring reads according to the reading frame in which they are found (as determined by the inferred A-site). This is what makes it clear that the read density in the first reading frame (red) is much higher within the ouORF which then decreases at the ouORF stop codon. The majority of tools used for visualizing Ribo-Seq data do not employ this technique making it much more difficult to visually identify dual coding regions. Trips-Viz also has the functionality to set a periodicity score, which filters out all reads with poor periodicity making the signal from the ouORF evident, saving users from having to identify and manually select studies with strong periodicity.

The ORF architecture beneath the subcodon profile in Figure 5 (horizontal red, green and blue bars) displays the positions of the AUG’s (short white lines) and stops (longer black lines). No AUG is visible in the first reading frame (red) that could act as a potential start for the ouORF. Trips-Viz, however, allows users to optionally enter any nucleotide sequence to be highlighted in the ORF architecture. In Figure 5 CUG codons are shown as short black lines in Frame 1 making it easier to see the exact position where the ouORF initiates. The merged CDS bar (dark blue bar just above the ORF architecture), shows all the regions of the transcript which overlap with other annotated CDS regions. As there is no dark blue bar in the non-overlapping region of the ouORF, it is possible to tell from this plot that the ouORF is not a part of the annotated (in the current annotation version) CDS in an alternative transcript without having to explore the exon architecture and annotation of the corresponding genomic locus.

The short code in brackets above the plot (14HD) can be used to recreate the plot in the browser using the same settings and files used to create the plot initially. Navigating to https://trips.ucc.ie/short/14HD in a browser will recreate the plot shown in Figure 5 and show which settings and files were used to generate the plot, as well as allowing users to make full use of the interactive features of the plot, like the ability to pan, zoom, turn on/off different plot elements, download the image as a high quality .png, and download the raw counts. Every plot created in Trips-Viz is linked to one of these short codes allowing them to easily be reproduced and shared. For example, navigating to https://trips.ucc.ie/short/14SJ will load a plot showing the same transcript isoform for *POLG* but with proteomics data, where sequences encoding peptides that match mass spectra are found. Their presence within the ouORF further strengthens the confidence that this ORF is translated and likely encodes a stable protein product. While performing a similar analysis on other platforms is certainly achievable, this example highlights the power and flexibility of Trips-Viz visualization. The combination of a large number of publicly available data with a versatile set of computational tools and visualizations makes such analyses both quick and easy.

## DATA AVAILABILITY

Trips-Viz is freely and publicly available at https://trips.ucc.ie with no login requirement. The source code for Trips-Viz is now also available on GitHub (https://github.com/skiniry/Trips-Viz) including instructions on how to setup a local instance of Trips-Viz.
